# Immune-Related Adverse Events and Their Association With the Effectiveness of PD-1/PD-L1 Inhibitors in Non-Small Cell Lung Cancer: A Real-World Study From China

**DOI:** 10.3389/fonc.2021.607531

**Published:** 2021-03-05

**Authors:** Xiaoling Chen, Jun Nie, Ling Dai, Weiheng Hu, Jie Zhang, Jindi Han, Xiangjuan Ma, Guangming Tian, Sen Han, Di Wu, Yang Wang, Jieran Long, Ziran Zhang, Jian Fang

**Affiliations:** Department of Thoracic Oncology II, Key Laboratory of Carcinogenesis and Translational Research (Ministry of Education/Beijing), Peking University Cancer Hospital and Institute, Beijing, China

**Keywords:** non-small cell lung cancer, immunotherapy, adverse events, real-world evidence, objective response, survival

## Abstract

**Background:**

Programmed cell death-1/programmed cell death ligand-1 (PD-1/PD-L1) inhibitors are increasingly used in China, but no real-world data are available about the immune-related adverse events (irAEs). This real-world retrospective study aimed to assess the safety and effectiveness of PD-1/PD-L1 inhibitors in patients with non-small cell lung cancer (NSCLC) and to analyze the association between irAEs and effectiveness.

**Methods:**

This was a retrospective study of the clinical data of patients with NSCLC treated with PD-1/PD-L1 inhibitors from August 2016 to November 2019 at Beijing Cancer Hospital. The patients were divided into the irAE or non-irAE groups. Overall adverse events, the impact of irAE on tumor response, and the association of irAEs with effectiveness were evaluated.

**Results:**

One hundred and ninety-one patients were included, including 70 (36.6%) patients in the irAE group and 121 (63.4%) patients in the non-irAE group. AE, grades 3–5 AEs, and irAE occurred in 107 (56.0%), 24 (12.6%), and 70 (36.6%) of the patients, respectively. The objective response rate (ORR) and disease control rate (DCR) were higher in the irAE group compared with the non-irAE group (42.0% vs. 25.8%, P=0.038; 91.9% vs. 70.8%, P=0.002). Multivariable analyses identified that irAE were associated with progression-free survival (HR=0.62, 95%CI: 0.43–0.91; P=0.015), but not with overall survival (HR=0.76, 95%CI: 0.44–1.28; P=0.299).

**Conclusion:**

In NSCLC treated with PD-1/PD-L1 inhibitors, patients with irAEs showed improved effectiveness over patients without irAEs. Future studies of anti-PD-1/PD-L1 immunotherapy should explore this association and the underlying biological mechanisms of efficacy.

## Introduction

Lung cancer is the leading cause of cancer-related death worldwide (1–3), with an incidence of 31.5 per 100,000 men and 14.6 per 100,000 women (3). It is mainly categorized as non-small cell lung cancer (NSCLC) and small cell lung cancer (SCLC) (4). NSCLC accounts for about 80% of all lung cancers, of which 75% of the patients are in an advanced stage at diagnosis (4).

In recent years, inhibitors of the programmed cell death 1 (PD-1)/programmed death-ligand 1 (PD-L1) have shown strong anti-tumor activity and become standard anti-tumor treatments for patients with lung cancer (5). To date, two PD-1 inhibitors (pembrolizumab and nivolumab) and one PD-L1 inhibitor (atezolizumab) have been approved for first- or second-line treatment of NSCLC by the US Food and Drug Administration (6, <x>7 </x>,^7^). Other PD-1/PD-L1 inhibitors (avelumab, camrelizumab, sintilimab, tislelizumab, toripalimab, camrelizumab, sintilimab, tislelizumab, and toripalimab) are at different stages of clinical development (8–11). Those drugs inhibit the immune escape mechanism of the tumor cells, allowing the body’s immune system to recognize the cancer cells as non-self and killing them (8–11).

The immune checkpoint inhibitors (ICIs), including the PD-1 axis inhibitors, activate the body’s immune system and can cause adverse events (AEs), including damaging the normal tissues and organs in the form of an inflammatory response, which is known as immune-related adverse events (irAEs) (12). Those irAEs can affect different organs and show different clinical symptoms such as skin (rash and pruritus), gastrointestinal (diarrhea and colitis), liver (hepatitis), endocrine (hyperthyroidism, hypothyroidism, and adrenal insufficiency), lung (pneumonitis), and kidney (renal insufficiency) (12). In addition, treatment-related adverse events (trAEs), including fatigue, anorexia, and nausea, are also among the adverse events of ICIs (13–15). In general, these toxicities are mild, but some specific irAEs can affect the treatment course, and can even be life-threatening (16).

At present, irAEs are thought to represent the bystander effect of activated T cells (e.g., due to a more competent/treatment-responsive immune system or cross-reactivity between tumor and host tissue) (17–19), and it is a reasonable assumption that the patients who respond to ICI are more likely to develop autoimmune toxicity. Previous studies have shown that irAE onset may represent one clinical biomarker for ICI response (20, 21). Several retrospective studies showed that irAEs were associated with durable response to ICIs and clinical benefit in patients with melanoma (22, 23), and several studies have shown similar associations with NSCLC treated with nivolumab (24–26). Nevertheless, to our knowledge, no similar studies have been reported for the treatment of PD-1/PD-L1 inhibitors (alone or in combination) in patients with advanced NSCLC in China.

Considering that PD-1/PD-L1 inhibitors are increasingly being used, it is important to fully understand their AEs in the treatment of NSCLC. Most of the current data on them come from clinical trials, which were mainly conducted in Caucasians, and only a small number of Asian participants were included in multi-ethnic trials. Therefore, the data about the AEs, especially irAEs of PD-1/PD-L1 inhibitors in the Chinese population, are not exhaustive. Therefore, the purpose of this study was to assess the incidence of irAEs and analyze the association of irAEs with the effectiveness of PD-1/PD-L1 inhibitors for patients with advanced NSCLC in the real-world Chinese population.

## Methods

### Patients

This was a retrospective study of patients with NSCLC treated with PD-1/PD-L1 inhibitors from August 2016 to November 2019 at the Second Department of Thoracic Medicine of Beijing Cancer Hospital. The study was approved by the Ethics Committee of Beijing Cancer Hospital (No. 2020YJZ24). The need for individual consent was waived by the committee because of the retrospective nature of the study.

The inclusion criteria were 1) cytological or histological confirmation of NSCLC (6, 27), 2) patients who received monotherapy with PD-1/PD-L1 inhibitors or PD-1/PD-L1 inhibitors with chemotherapy or PD-L1 plus CTLA-4 with or without chemotherapy, or any other regimen that includes PD-1/PD-L1 inhibitors, 3) completed at least one cycle of immunotherapy, and 4) available data about the AEs. The exclusion criteria were 1) important organ dysfunction before treatment or 2) did not complete an AE follow-up visit of one cycle at the time of data collection.

### Data Collection

Patients’ data were collected through the information system of Beijing Cancer Hospital (HIS), which is a comprehensive electronic patient chart system that is fully indexed and searchable (28). Clinical data included age, sex, pathological type, Eastern Cooperative Oncology Group (ECOG) score, weight change before treatment, smoking history, driver gene variants [epidermal growth factor receptor (EGFR)/anaplastic lymphoma kinase (ALK)], tumor-node-metastasis (TNM) stage, line of immunotherapy, and other basic clinical characteristics as well as medication regimen, adverse events, clinical effectiveness, and prognosis were recorded. All patients routinely underwent preoperative systematic physical examination, complete blood count, and routine biochemical examination. Weight loss was defined as a weight loss >5% within 6 months.

### AEs and Effectiveness Evaluation

AEs were judged according to the National Cancer Institute Common Terminology Criteria for Adverse Events (NCI-CTCAE) version 4.03 (29) and were classified as grades I–V. irAEs were defined according to the guidelines on the management of immunotherapy-related toxicities (12, 30). The time to onset of an irAE was defined as the time from the start of immunotherapy to the occurrence of irAE. irAEs were defined as having a potential immunological basis that required more frequent monitoring and potential intervention. Based on this, the patients were divided into two groups (the irAE and non-irAE groups), and the overall response rate (ORR), progression-free survival (PFS), and overall survival (OS) were evaluated in each group.

According to the Response Evaluation Criteria in Solid Tumors (RECIST) version 1.1 (31), the response was divided into the complete response (CR), partial response (PR), stable disease (SD), and progressive disease (PD). The ORR was (CR+PR)/(CR+PR+SD+PD) ×100%. The disease control rate (DCR) was (CR+PR+SD)/(CR+PR+SD+PD) ×100%. Target and non-target lesions were measured routinely by radiologists and effectiveness were evaluated routinely by physician internists as part of the routine clinical workup. In the present study, the assessment was performed unblinded and retrospectively by two attending physicians with >8 years of experience and two associate physicians with >3 years of experience in medical oncology.

The PFS was defined as the time from the start of treatment to progression or death from any cause. The OS was defined as the time from the start of treatment to death from any cause. The values of the patients who were lost to follow-up, and those who did not progress were treated as censored values. The censored time was the last follow-up that confirmed that the patients had neither progressed nor died.

### Outcomes and Follow-Up

The outcome of the study was the effectiveness of PD-1/PD-L1 inhibitors, including ORR, PFS, and OS. The effectiveness of the 3-week regimens was assessed every 6 weeks, and that of the 2-week regimens every 8 weeks. Thyroid function, myocardial enzymes, B-type brain natriuretic peptide (BNP), lipase, and amylase were examined before the first treatment and every 3 months. After completing the treatment, the patients were followed for progression or survival every 3 months by clinical visits and telephone interviews. In this study, there were three researchers responsible for follow-up. They all have participated in nearly eight clinical trials of PD-1/PD-L1 inhibitor treatment as sub-investigators, and they received training to identify AE and irAE. Besides, one person, a staff of the Statistics Department of the Beijing Cancer Hospital with 15-year-experience, was responsible for collecting the survival data of the patients. The follow-up was censored on May 27, 2020.

### Statistical Analysis

SPSS 23.0 (IBM, Armonk, NY, USA) and GraphPad Prism 8 (GraphPad Software, San Diego, CA, USA) were used for statistical analysis and graph plotting. The Kolmogorov-Smirnov test was applied to assess the normal distribution of continuous data. The continuous data are presented as medians (ranges) and were analyzed using the Mann-Whitney U-test. The categorical data are presented as numbers (percentages) and were analyzed using the chi-square test or Fisher’s exact test. The time to the onset of irAE (grades 1–2 vs. grades 3–5) was compared using the Kruskal-Wallis rank-sum test. The Kaplan-Meier method and the log-rank test were used for comparison of survival data between groups. The univariable and multivariable Cox regression model was used for the analysis of PFS and OS. The data filtering method used in the multivariable Cox regression was backward stepwise (likelihood ratio). Factors with P<0.10 in the univariable analyses and factors with unbalanced baseline characteristics were included in the multivariable analysis. P-values <0.05 were considered statistically significant.

## Results

### Characteristics of the Patients

A total of 222 patients were screened: 191 were included, and 31 were excluded (the treatment (active vs. placebo) could not be confirmed in 19 patients due to inclusion in a randomized, double-blind clinical trial; six patients were enrolled in phase I clinical trials to receive combined anti-PD-1 and its downstream target double-inhibitor; two patients had an abnormal liver function; one patient had abnormal kidney function before treatment; three patients did not complete the evaluation of adverse reactions after the first cycle).

The characteristics of the patients are shown in **Table 1**. The 191 patients were divided into two groups: the irAE group (70, 36.6%) and the non-irAE group (121, 63.4%). The median age was 62 years (range, 30–87). There were no differences between the two groups in terms of sex, NSCLC histology, ECOG, changes in weight before treatment, smoking history, stage, EGFR status, and ALK status. There were differences between patients with or without irAE, including age, the lines of immunotherapy, type of drug, and therapeutic modalities (all P<0.05). Treatment drugs and treatment cycles were summarized in **Supplementary Table S1**. Among the 191 patients, 69 patients participated in clinical trials.

**Table 1:** Characteristics of the patients.

Characteristics, N (%)		irAE Group (n=70)	Non-irAE Group (n=121)	P
Sex	Male	54 (38.8)	85 (61.2)	0.302
	Female	16 (30.8)	36 (69.2)	
Age (years)	<65	52 (41.9)	72 (58.1)	0.039
	≥65	18 (26.9)	49 (73.1)	
NSCLC histology	Adenocarcinoma	41 (38.0)	67 (62.0)	0.497
	Squamous carcinoma	27 (37.5)	45 (62.5)	
	Others	2 (18.2)	9 (81.8)	
ECOG	0–1	68 (38.2)	110 (61.8)	0.138
	≥2	2 (15.4)	11 (84.6)	
Changes in weight before treatment	No change	55 (37.4)	92 (62.6)	0.688
	Loss of weight	15 (34.1)	29 (65.9)	
Smoking history	Yes	27 (38.0)	44 (62.0)	0.945
	No	42 (36.2)	74 (63.8)	
	Unknown	1 (25.0)	3 (75.0)	
Stage	I	1 (20.0)	4 (80.0)	0.675
	II	4 (25.0)	12 (75.0)	
	III	17 (34.0)	33 (66.0)	
	IV	47 (39.8)	71 (60.2)	
	Unknown	1(50)	1 (50)	
EGFR status	Wild type	46 (38.7)	73 (61.3)	0.746
	Mutant	5 (31.3)	11 (68.8)	
	Unknown	19 (33.9)	37 (66.1)	
ALK status	Wild type	55 (38.7)	87 (61.3)	0.614
	Fusion	0 (0)	1 (100.0)	
	Unknown	15 (31.3)	33 (68.8)	
Lines of immunotherapy	First line	39 (52.0)	36 (48.0)	0.005
	Second line	15 (27.3)	40 (72.7)	
	Third line and above	11 (24.4)	34 (75.6)	
	Others	5 (31.3)	11 (68.7)	
Type of drug	Anti-PD-1	43 (27.9)	111 (72.1)	<0.001
	Anti-PD-L1	27 (73.0)	10 (27.0)	
Therapeutic modalities	Single drug	29 (29.3)	70 (70.7)	0.019
	Combined with chemotherapy	35 (42.7)	47 (57.3)	
	Combined with CTLA-4	5 (83.3)	1 (16.7)	
	Others	1 (25.0)	3 (75.0)	

irAE, immune-related adverse events; NSCLC, non-small cell lung cancer; ECOG, Eastern Cooperative Oncology Group; EGFR, epithelial growth factor receptor; ALK, anaplastic lymphoma kinase; PD-1, programmed cell death protein 1; PD-L1, programmed cell death protein ligand 1; CTLA-4, cytotoxic T-lymphocyte-associated protein 4. P-values <0.05 were considered statistically significant.

### Adverse Events

As shown in **Table 2**, AE, grades 3–5 AEs, and irAE were occurred in 107 (56%), 24 (12.6%), and 70 (36.6%) of the 191 patients, respectively.

**Table 2:** Overall adverse events.

AE, N (%)	All AEs (n=191)	Grades 3–5 AEs (n=191)
Total	107 (56.0)	24 (12.6)
Poor appetite	28 (14.7)	1 (0.5)
Fatigue	27 (14.1)	0
Nausea	24 (12.6)	1 (0.5)
Fever	20 (10.5)	1 (0.5)
Pneumonia	19 (9.9)	5 (2.6)
Vomiting	12 (6.3)	1 (0.5)
Influenza-like symptoms	6 (3.1)	0
Dizziness	5 (2.6)	0
Reactive capillary hemangiomas	5 (2.6)	0
Headache	4 (2.1)	1 (0.5)
Stomachache	4 (2.1)	0
Mucosal ulcer	4 (2.1)	0
Transfusion reaction	3 (1.6)	0
Dry mouth	2 (1.0)	0
Dysphagia	2 (1.0)	1 (0.5)
Hyperglycemia	2 (1.0)	1 (0.5)
irAE	70 (36.6)	14 (7.3)
Rash	21 (11.0)	0
Pruritus	16 (8.4)	0
Pneumonitis	15 (7.9)	4 (2.1)
ALT increase	14 (7.3)	2 (1.0)
Hypothyroidism	9 (4.7)	1 (0.5)
Creatinine increased	8 (4.2)	3 (1.6)
Diarrhea	8 (4.2)	0
AST increased	8 (4.2)	1 (0.5)
GGT increased	7 (3.7)	2 (1.0)
Myalgia	6 (3.1)	0
Arthralgia	6 (3.1)	1 (0.5)
Hyperthyroidism	5 (2.6)	0
Amylase increase	5 (2.6)	1 (0.5)
Elevated muscle enzymes	3 (1.6)	1 (0.5)
Lipase increase	3 (1.6)	2 (1.0)
Bilirubin increased	2 (1.0)	0
Pancreatitis	2 (1.0)	0
Neurotoxicity^a^	1 (0.5)	0
Hypophysitis^b^	1 (0.5)	1 (0.5)
Hepatitis	1 (0.5)	1 (0.5)

a One patient with neurotoxicity presented with a drooping eyelid.

b Only one drug-related death was hypophysitis.

AE, adverse event; irAE, immune-related adverse events; ALT, alanine transaminase; AST, aspartate transaminase; GGT, γ-glutamyltransferase.

Among all 191 patients enrolled in this study, the most common overall AEs were poor appetite (14.7%), fatigue (14.1%), nausea (12.6%), and fever (10.5%). The endocrine AEs were mainly hypothyroidism (4.7%), hyperthyroidism (2.6%), and hypophysitis (0.5%). The gastrointestinal toxicities were diarrhea (4.2%) and immune-related pancreatitis (1.0%). Nervous system and musculoskeletal toxicities included drooping eyelids (0.5%), myalgia (3.1%), and arthralgia (3.1%).

In all patients enrolled in this study, the most common irAEs were rash (11.0%), pruritus (8.4%), immune-related pneumonitis (7.9%), and elevated ALT (7.3%). The most common grades 3–5 irAEs were immune-related pneumonitis (2.1%), increased creatinine (1.6%), increased GGT (1.0%), increased ALT (1.0%), and increased lipase (1.0%). Most irAEs were mild, and the incidence of grades 3–5 irAEs was low. Only one drug-related death (hypophysitis) was observed. The patient who developed grade 5 hypophysitis was a 72-year-old man treated with pembrolizumab (2 mg/kg q 3 weeks). After five cycles, he developed hypophysitis that showed as drowsiness, fatigue, consciousness disorder, and hypotension. Laboratory examination showed mild hyponatremia, secondary adrenal insufficiency, and secondary hypothyroidism. Steroid treatment was ineffective. The patient also had immune-related pneumonitis.

The median time to onset of irAEs was shown in **Supplementary Figure S1**. As shown in **Supplementary Figure S2**, grades 1–2 irAEs occurred earlier than grades 3–4 irAEs (P=0.005).

Among the patients with irAE, 19 were treated with steroids (13 with immune-related pneumonitis, two with immune-related pancreatitis, one with elevated amylase and lipase, one with immune-related hypophysitis combined with immune-related pneumonitis, one with immune-related hepatitis, and one with elevated creatinine), and one patient with immune-related pneumonitis was treated with low-dose cyclophosphamide. No Patients were treated with infliximab or tocilizumab. Six patients were treated with thyroid hormone replacement.

This study included four patients with abnormal liver function and one patient with abnormal renal function at baseline. After immunotherapy, there was no aggravation of abnormal liver function or renal function. A grade 2 rash was observed in a patient with psoriasis (28 days after the combination of PD-L1 inhibitor + CTLA-4 inhibitor), a grade 2 immune-related pneumonitis was observed in one patient with rheumatoid arthritis (68 days after starting the combination of chemotherapy and PD-L1 inhibitor), and one patient presented with a tuberculosis relapse (178 days after chemotherapy combined with PD-L1 inhibitor).

### Association Between irAEs and Effectiveness

The last follow-up time was in May 2020. The average follow-up time was 9.8 months, and the longest follow-up time was 43.5 months. There were 175 cases of stage IV or recurrent NSCLC among those patients: 64 in the irAE group and 111 in the non-irAE group.

As shown in **Table 3**, there were 191 cases of NSCLC, and the response was evaluated in 151 patients: none had CR, 49 (32.5%) had PR, 71 (47.0%) had SD, and 31 (20.6%) had PD; ORR was 32.5%, and DCR was 80.1%. The CR, PR, SD, and PD rates of the irAE and non-irAE groups were 0, 42.0%, 50.0%, and 8.0% vs. 0, 25.8%, 44.9%, and 29.2%, respectively (P=0.002). The ORR and DCR rates of the irAE were higher than in the non-irAE group (42.0% vs. 25.8%, P=0.038; 91.9% vs. 70.8%, P=0.002).

**Table 3:** Impact of irAE on tumor response.

Effectiveness of treatment	All (N = 151)	irAE Group (n = 62)	non-irAE Group (n = 89)	P
CR	0	0	0	0.002
PR	49 (32.5%)	26 (42.0%)	23 (25.8%)	
SD	71 (47.0%)	31 (50.0%)	40 (44.9%)	
PD	31 (20.6%)	5 (8.0%)	26 (29.2%)	
ORR%	49 (32.5%)	26 (42.0%)	23 (25.8%)	0.038
DCR%	120 (80.1%)	57 (91.9%)	63 (70.8%)	0.002

irAE, immune-related adverse events; CR, complete response; PR, partial response; SD, stable disease; PD, progressive disease; ORR, objective response rate; DCR, disease control rate.

P-values <0.05 were considered statistically significant.

**Figure 1:**
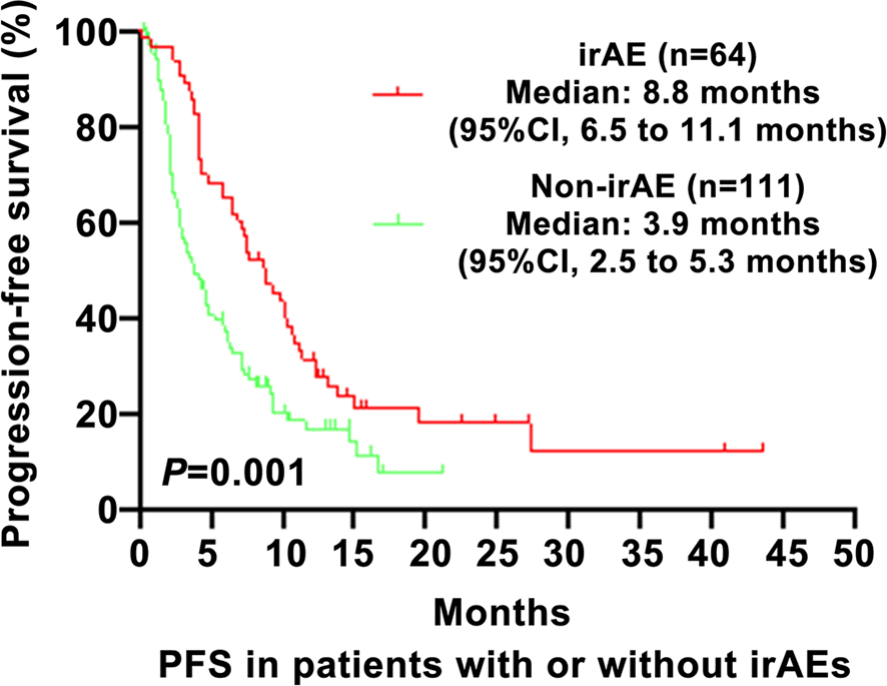
Impact of immune-related adverse events (irAEs) on progression-free survival (PFS). PFS, progression-free survival; CI, confidence interval; irAE, immune-related adverse events; P-values <0.05 were considered statistically significant.

**Figure 2:**
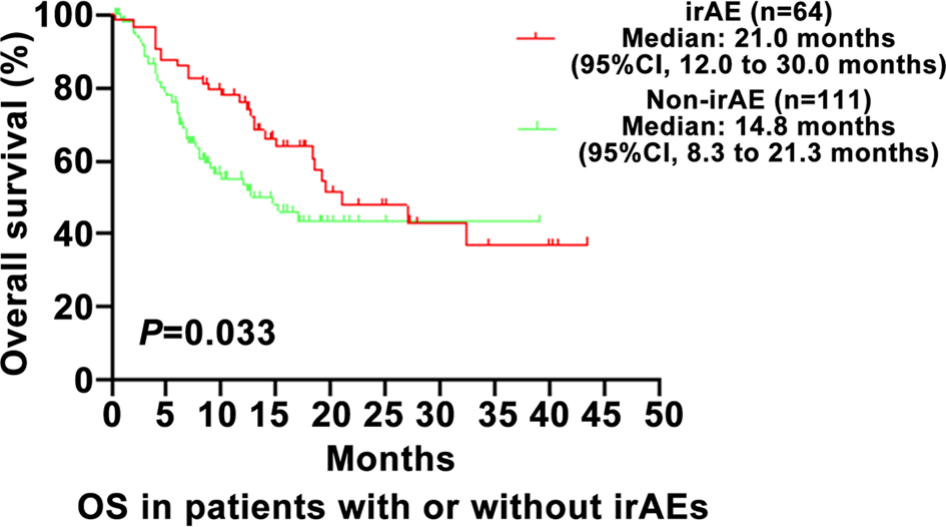
Impact of immune-related adverse events (irAEs) on overall survival (OS). OS, overall survival; CI, confidence interval; irAE, immune-related adverse events; P-values <0.05 were considered statistically significant.

As shown in **Figure 1**, the PFS of the irAE group was longer than in the non-irAE group in advanced NSCLC (8.8 vs. 3.9 months, 95% CI: 6.5–11.1 vs. 2.5–5.3, P=0.001). As shown in **Figure 2**, the OS of the irAE group was longer than the non-irAE group in advanced NSCLC (21.0 vs. 14.8 months, 95% CI:12.0–30.0 vs. 8.3–21.3, P=0.033).

As shown in **Table 4**, the univariable analysis suggested that age, ECOG, EGFR status, lines of immunotherapy, type of drug, therapeutic modalities, and irAE might be prognostic factors of PFS (all P<0.10). And the multivariable analysis of advanced NSCLC showed that ECOG≥2 (HR=1.97, 95%CI: 1.03–3.76, P=0.04), second line of immunotherapy (vs. first line, HR=1.75, 95%CI: 1.15–2.65, P=0.008) and irAE (HR=0.62, 95%CI: 0.43–0.91, P=0.015) were independent associated with PFS.

**Table 4:** Univariate and multivariable analyses of factors associated with PFS.

Factor	Univariable		Multivariable	
	HR (95% CI)	P	HR (95% CI)	P
Male	0.88 (0.60–1.29)	0.513		
Age ≥65 years	1.54 (1.08–2.20)	**0.018**	1.37 (0.95–1.97)	0.091
NSCLC histology				
Adenocarcinoma	1			
Squamous	0.88 (0.61–1.27)	0.498		
Other	1.13 (0.52–2.45)	0.764		
ECOG ≥2	2.51 (1.34–4.71)	**0.004**	1.97 (1.03–3.76)	**0.04**
No change in weight before treatment	1.15 (0.92–1.43)	0.216		
Smoking history				
No	1			
Yes	0.92 (0.64–1.32)	0.64		
Unknown	1.36 (0.49–3.77)	0.555		
EGFR status				
Wild type	1		1	
Mutant	1.89 (1.03–3.47)	**0.039**	1.76 (0.93–3.31)	0.082
Unknown	0.86 (0.57–1.31)	0.491	0.86 (0.56–1.30)	0.469
ALK status				
Wild type	1			
Fusion	0.00 (0.00–1.25E162)	0.959		
Unknown	0.73 (0.48–1.12)	0.154		
Lines of immunotherapy				
First line	1		1	
Second line	1.99 (1.32–3.00)	**0.001**	1.75 (1.15–2.65)	**0.008**
Third line and above	1.64 (1.06–2.53)	**0.026**	1.43 (0.91–2.25)	0.119
Type of drug, Anti-PD-L1	0.56 (0.36–0.87)	**0.01**	0.73 (0.45–1.19)	0.204
Therapeutic modalities				
Single drug	1		1	
Combined with chemotherapy	0.73 (0.51–1.04)	**0.083**	0.82 (0.48–1.39)	0.459
Combined with CTLA-4	0.79 (0.34–1.84)	0.588	1.82 (0.69–4.82)	0.23
Others	0.32 (0.04–2.27)	0.252	0.34 (0.04–2.65)	0.305
irAE	0.54 (0.37–0.77)	**0.001**	0.62 (0.43–0.91)	**0.015**

PFS, progression-free survival; HR, hazard ratio; CI, confidence interval; irAE, immunotherapy-related adverse events; ECOG, Eastern Cooperative Oncology Group; EGFR, epithelial growth factor receptor; ALK, anaplastic lymphoma kinase; Bold values indicate P-values <0.05, considered statistically significant.

As shown in **Table 5**, the univariable analysis suggested that age, ECOG, smoking history, lines of immunotherapy, type of drug, and irAE might be prognostic factors of OS (all P<0.10). And the multivariable analysis of advanced NSCLC showed that age ≥65 years (HR=1.81, 95%CI: 1.13–2.88, P=0.013) and ECOG≥2 (HR=4.92, 95%CI: 2.40–10.05, P<0.001) were independent associated with OS.

**Table 5:** Univariable and multivariable analyses of factors associated with OS.

Factor	Univariable		Multivariable	
	HR (95% CI)	P	HR (95% CI)	P
Male	1.27 (0.76–2.11)	0.364		
Age ≥65 years	2.08 (1.32–3.26)	**0.001**	1.81 (1.13–2.88)	**0.013**
NSCLC histology				
Adenocarcinoma	1			
Squamous	1.02 (0.63–1.63)	0.945		
Others	0.85 (0.30–2.35)	0.747		
ECOG ≥2	6.15 (3.06–12.37)	**<0.001**	4.92 (2.40–10.05)	**<0.001**
No change in weight before treatment	0.91 (0.70–1.18)	0.469		
Smoking history				
No	1		1	
Yes	1.26 (0.78–2.02)	0.345	1.31 (0.79–2.18)	0.302
Unknown	2.60 (0.91–7.45)	**0.075**	1.13 (0.37–3.48)	0.829
EGFR status				
Wild type	1			
Mutant	0.68 (0.27–1.70)	0.408		
Unknown	0.87 (0.51–1.48)	0.599		
ALK status				
Wild type	1			
Fusion	0.00 (0.00–1.46E225)	0.967		
Unknown	0.76 (0.43–1.33)	0.334		
Lines of immunotherapy				
First line	1		1	
Second line	1.62 (0.96–2.71)	**0.069**	1.31 (0.74–2.32)	0.360
Third line and above	1.19 (0.68–2.10)	0.548	0.95 (0.46–1.97)	0.883
Type of drug, Anti-PD-L1	0.51 (0.28–0.94)	**0.03**	0.61 (0.32–1.13)	0.117
Therapeutic modalities				
Single drug	1		1	
Combined with chemotherapy	0.68 (0.42–1.09)	0.111	0.72 (0.43–1.19)	0.197
Combined with CTLA-4	0.97 (0.35–2.70)	0.95	1.70 (0.54–5.40)	0.367
Others	0.00 (0.00–3.45E267)	0.97	0.00 (0.00–2.10E222)	0.964
irAE	0.60 (0.37–0.97)	**0.036**	0.76 (0.44–1.28)	0.299

OS, overall survival; HR, hazard ratio; CI, confidence interval; irAE, immunotherapy-related adverse events; ECOG, Eastern Cooperative Oncology Group; EGFR, epithelial growth factor receptor; ALK, anaplastic lymphoma kinase; Bold values indicate P-values <0.05, considered statistically significant.

Sixty-five percent of all irAEs in the patients in this study occurred within 3 months. The median time to the first irAE was 1 month. Therefore, to account for a possible immortal time bias, the patients were divided into five groups: ICI >1 month, non-irAE; ICI <1 month, non-irAE; ICI >1 month, irAE <1 month; ICI <1 month, irAE <1 month; and ICI >1 month, irAE >1 month. The PFS was different for all patients (P<0.001) and ICI >1 month (P=0.006), but not for ICI <1 month (P=0.747). The OS was different for all patients (P<0.001), but not for ICI >1 month (P=0.072) and ICI <1 month (P=0.456).

### Analysis of Association in Different Status

The differences in PFS and OS between the irAE and non-irAE groups according to specific organs (skin, pneumonitis, hepatotoxicity, and thyroid dysfunction) were not statistically significant (**Supplementary Figure S3**). In this study, 17 patients discontinued PD-1/PD-L1 inhibitors due to irAE among 175 patients with metastasis or recurrence, including eight patients with PR and nine patients with SD. The median PFS from treatment initiation was 13.9 months for PR patients and 12.4 months for SD patients (P=0.301) (**Supplementary Figure S4A**). The median PFS from the discontinuation was 11.1 months for PR patients and 5.1 months for SD patients (P=0.279) (**Supplementary Figure S4B**). The median OS from the initiation was NR for PR or SD patients (P=0.04) (**Supplementary Figure S4C**). The median OS from the discontinuation was NR for PR and 9.9 months for SD patients (P=0.041) (**Supplementary Figure S4D**). There were 64 patients with irAE in this study, 17 of whom were treated with steroid therapy for irAE. PFS was 4.2 (95%CI: 2.3–6.2) months in the patients with steroid use and 9.3 (95%CI: 6.7–11.9) months in those without (P=0.291) (**Supplementary Figure S5A**). OS was 12.4 (95%CI: 4.0–20.8) months in steroid-treated patients and 27.0 (95%CI: 9.4–44.6) months in non-steroid-treated patients (P=0.005) (**Supplementary Figure S5B**). After adjusting for the type of drug, therapeutic modalities, lines of immunotherapy, age, smoking history, EGFR status, and ECOG, the multivariate Cox analysis showed that steroid use was independently associated with OS (HR=2.86, 95%CI: 1.30–6.30, P=0.009) but not with PFS (HR=1.34, 95%CI: 0.69–2.59, P=0.386).

### Subgroup Analysis in the Patients With Stage IV

The overall number of therapy lines were 1–8 lines (mean of 2.3 lines) and 1–8 lines (mean of 2.7 lines) in the irAE and non-irAE groups of stage IV patients, respectively. Among the 118 stage IV patients, 47 were in the irAE group, and 71 were in the no-irAE group. The median progression-free survival (PFS) was 8.7 (95% CI: 7.0–10.4) months in the irAE group and 3.9 (95% CI: 2.4–5.4) months in the non-irAE group (P=0.002) (**Supplementary Figure S6A**). The median overall survival (OS) was 27.0 (95%CI: 13.4–40.6) months in the irAE group and 14.8 (95%CI: 10.7–18.9) months in the non-irAE group (P=0.069) (**Supplementary Figure S6B**).

## Discussion

PD-1/PD-L1 inhibitors are increasingly used in China, but few real-world data are available about the irAEs to our knowledge. This real-world retrospective study aimed to assess the safety and effectiveness of PD-1/PD-L1 inhibitors in patients with NSCLC and to analyze the association between irAEs and effectiveness. The results suggest that the TRAEs of PD-1/PD-L1 inhibitors in NSCLC were generally of low grade. irAEs presented mainly as skin toxicity, immune-related pneumonitis, and hepatotoxicity. irAEs were associated with higher ORR and DCR and longer PFS. To the best of our knowledge, this study on the irAEs and their association with the effectiveness of PD-1/PD-L1 inhibitors in NSCLC is by far the one with the largest number of patients in the real-world NSCLC Chinese population.

Approximately 56.0% of the patients developed AEs, and 36.6% developed irAEs among all 191 patients in this study. The most common AEs were poor appetite, fatigue, nausea, and fever. The most common grades 3–5 treatment-related AE was pneumonia (2.6%). The most common irAEs were rash and pruritus, immune-related pneumonitis, and increased ALT. The most common grades 3–5 irAEs were immune-related pneumonitis (2.1%), increased creatinine (1.6%), increased GGT (1.0%), increased ALT (1.0%), and increased lipase (1.0%). The intensity of irAE was generally mild to moderate, with only 7.3% of patients with grades 3–5. The type of irAEs observed in this study was similar to those observed in the previous studies about PD-1/PD-L1 inhibitors, but the incidence of immune-related pneumonitis was higher than that reported in previous studies (13, 32, 33). This might be due to the selection criteria used in the various trials. Nevertheless, no new safety signal was observed.

Among the 191 patients in this study, the most common irAEs were rash and pruritus, immune-associated pneumonitis, and increased ALT. The most common grades 3–5 irAEs were immune-associated pneumonitis, increased creatinine, increased GGT, and increased ALT. Immune-associated pneumonitis is a potentially lethal irAE, and it is a focus of attention among irAEs of lung cancer. In this study, the incidence of immune-associated pneumonitis was 7.9%, and the incidence of grades 3–5 was 2.1%. This was more frequent than in a previous study (33). The possible reason might be that patients with basic lung diseases (such as chronic interstitial bronchitis) were excluded from the clinical trials. In addition, many previous studies of AEs included a variety of tumors (melanoma, kidney cancer, and non-small cell lung cancer). Because the microenvironment of lung cancer is different from that of other tumors, the incidence of lung cancer immune-associated pneumonitis could be slightly higher. Third, this study included patients treated with PD-1/PD-L1 combined with other drugs, which might increase the likelihood of developing immune-related pneumonitis. There were no deaths due to immune-related pneumonitis in this study, and it could usually be relieved by timely detection and standard treatment. Skin toxicity was the most common irAEs in all patients enrolled in this study, mainly rash (11.0%) and pruritus (8.4%) of grades 1–2. This is consistent with previous studies (13, 32). Hepatotoxicity was another of the most common irAE, with an overall incidence of 7.3% (increased ALT), and the incidence of grades 3–5 was 1.0% (increased ALT or GGT). The higher incidence compared with previous studies might be related to the combined medication regimens included in this study. Endocrine toxicity mainly manifested as hypothyroidism (4.7%) and hyperthyroidism (2.6%), with only one case of grades 3–5 hypothyroidism, similar to previous researches (13, 34). Uncommon irAEs also occurred in our study: two cases of pancreatitis, one of neurotoxicity, and one of hypophysitis. The proportion of pneumonia was high (9.9% of any-grade and 2.6% of grades 3–5), which is significantly higher than the incidence rate during chemotherapy. During PD-1/PD-L1 combined with chemotherapy, the decrease of leukocytes and neutrophils may increase the risk of pneumonia during immunotherapy, and it needs to be clinically distinguished from immune-related pneumonitis.

Although the precise mechanisms of irAEs have not been fully revealed, they are thought to be the bystander effect of activating T cells, which is consistent with the mechanism of ICIs (12). irAEs might be triggered by antigens that are common to both tumor and inflamed organs. Second, the link between T-cells and irAEs focuses on the gut microbiome, and significant differences in microbial diversity might be observed in responding versus non-responding patients. Third, pre-existing organ-specific antigen expression may be another cause of irAEs without representing a shared effect from anti-tumor activity, which could be mechanisms of autoimmune toxicity that are independent of the anti-tumor response (20). irAE onset may be a clinical biomarker for the response of immune checkpoint blocking drugs. This phenomenon was first seen in melanoma patients, although not all evidence supports this hypothesis. Several recent retrospective analyses showed that among patients receiving nivolumab, patients with irAE had a better response (ORR or DCR or PFS or OS) than patients without irAE (22–24, 35, 36).

The patients with irAE had higher ORR and DCR and a longer median PFS, but not OS. It is suggested that irAE may be a clinical biomarker for the benefit of immunotherapy, including PD-1/PD-L1 inhibitors. It reminds the medical team to monitor and detect irAE in time in order to reduce or avoid the occurrence of serious irAE, thereby reducing the proportion of termination and suspension of treatment. Some previous studies suggested that skin toxicity and thyroid function damage were related to the effectiveness of immunotherapy (37, 38). Previous studies suggested that specific types of irAEs were related to prognosis (39–43), but this was not observed here. In addition, cancers with a high tumor mutational burden (TMB) are associated with a higher risk of irAEs, and the possible cause is the different neoantigenic load across cancer types (44), but TMB could not be examined in this study. In this study, 17 patients discontinued treatments due to irAEs; among them, those with PR at discontinuation had a longer OS than those with SD, as supported by a previous study (45). Steroid use also had an impact on survival, as suggested by a previous study (46). The relationship between different irAEs and immunotherapy needs to be confirmed by a larger number of studies. In the future, prospective research is needed to verify the exact impact of specific irAEs on prognosis.

The median age (65 years old) was used as the cut-off, and there was a significant difference between <65 and ≥65 years in the irAE rates. That might be caused by slightly more patients under 65 years of age received a four-drug combination therapy (chemotherapy + immunotherapy + anti-angiogenic therapy) than patients over 65 years of age, and some patients over 65 years of age were given immunotherapy combined with anti-vascular therapy without chemotherapy in our clinical practice.

PD-1/PD-L1 inhibitors are increasingly used in China, but no real-world data are available. This study revealed that the irAEs of PD-1/PD-L1 inhibitors (either as monotherapy or combination therapy) for lung cancer were mainly low grade and suggested that patients with irAEs showed improved effectiveness over patients without irAEs. These data come from real-world results from the Chinese population, so it could better reflect the impact of the irAEs in the actual clinical practice. In addition, this study explored the correlation between the time of occurrence of irAEs or irAEs in different organs with the prognosis and provided data and clues for an in-depth study of the relationship between irAE and prognosis. This study provides a theoretical basis for the clinical use of PD-1/PD-L1 inhibitors in the Chinese population and provides clues for exploring the mechanism of the association of irAEs with effectiveness.

In this study, the patients with elevated creatinine as irAE had no combined medications and related disease history that could explain the renal damage. They included the patients treated with ICI monotherapy and patients treated with combined chemotherapy who did not display elevated creatinine during combined chemotherapy. Patients with elevated creatinine in the maintenance phase were considered to be more likely to be caused by immunotherapy. Nevertheless, because these patients did not undergo a kidney biopsy, the side effects of chemotherapy cannot be excluded, which is a limitation of this study. Besides, patients with pneumonia had clear evidence of infection, including elevated WBC, elevated NE%, high PCT, high CRP, or conditional pathogens from sputum culture or blood culture, and patients who improved after antibiotic treatment. The patients with pneumonitis had no clear clinical evidence of infection (hematological examination and pathogenic bacteria), and interstitial pneumonitis was considered in the imaging or patients who are ineffective in antibiotic therapy and get better after hormone therapy.

This study has limitations. First, this study is a retrospective study with data offsets. Second, at present, there is no clear diagnostic standard for irAE, and some of them are clinical symptoms that might be subjective. The present study determined irAE based on previous research and guidelines (12). There might be deviations in irAE determination. Because of the retrospective nature of the study, the exact timing of irAE occurrence (early vs. late) could not be determined with any accuracy and could not be examined against survival, but this relationship has been described (24).

In conclusion, we found that irAEs of PD-1/PD-L1 inhibitors for lung cancer were mainly low grade and that the occurrence of irAE was positively correlated with ORR, DCR, and PFS, suggesting that patients with irAEs are more likely to benefit from immunotherapy. These data come from real-world results from the Chinese population, including patients with some previous autoimmune diseases, tuberculosis, chronic bronchitis, and ECOG grade 2/3, so it could better reflect the irAE performance in the actual clinical practice environment. Future prospective studies are needed to confirm those results and explore the mechanism of irAEs’ association with effectiveness.

## Data Availability Statement 

The original contributions presented in the study are included in the article/**Supplementary Material**. Further inquiries can be directed to the corresponding author.

## Ethics Statement 

The studies involving human participants were reviewed and approved by the ethics committee of Beijing Cancer Hospital. Written informed consent for participation was not required for this study in accordance with the national legislation and the institutional requirements.

## Author Contributions

XC and JF contributed to the conception, design, analysis of data, and drafted the manuscript. JN, LD, and WH participated in the design of the study and acquisition of data. JZ, JH, XM, GT, SH, DW, YW, JL, and ZZ contributed to the acquisition of data. All authors contributed to the article and approved the submitted version.

## Funding

This work was part of the Program on the establishment of a comprehensive evaluation system for the efficacy of immunologic checkpoint inhibitors in the treatment of advanced non-small cell lung cancer funded under the Capital’s Funds for Health Improvement and Research, grant number: 2020-2-2153. Financial support for this study was received from Special Funds for Clinical Research of Wu Jieping Medical Foundation, grant number: 320.6750.19088-66.

## Conflict of Interest

The authors declare that the research was conducted in the absence of any commercial or financial relationships that could be construed as a potential conflict of interest.
