# Supplementary material for: Immune-Related Adverse Events and Their Association With the Effectiveness of PD-1/PD-L1 Inhibitors in Non-Small Cell Lung Cancer: A Real-World Study From China
Source: Front Oncol. 2021 Mar 5;11:607531. doi: 10.3389/fonc.2021.607531 (PMC7973369; doi:10.3389/fonc.2021.607531)
Supplement: Supplementary file 1 [file DataSheet_1.docx]

Supplementary Material

# Supplementary Figures and Tables

## Supplementary Figures

**Supplementary Figure S1.** Time to onset of immune-related adverse events (irAE).

**Supplementary Figure S2.** Time to onset of different grades (1-2 vs. 3-5) of immune-related adverse events (irAE). The data are presented as median (bold line) and range (box). P=0.005.


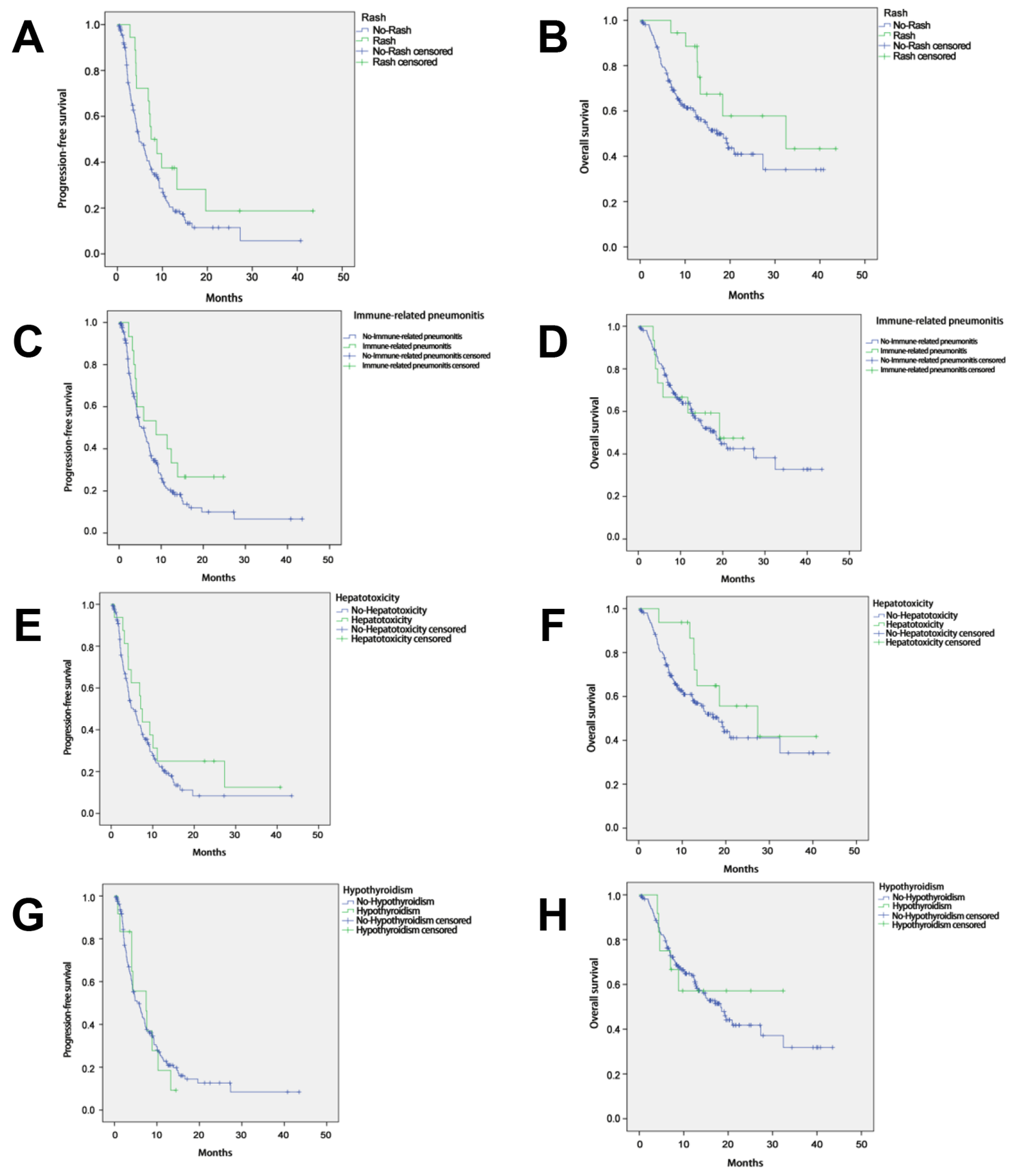


**Supplementary Figure S3.** Comparisons of progression-free survival (PFS) and overall survival (OS) in different irAEs patients. (A-B) PFS and OS according to skin irAEs. (C-D) PFS and OS, according to immune-related pneumonitis. (E-F) PFS and OS, according to hepatotoxicity. (G-H) PFS and OS according to thyroid dysfunction.


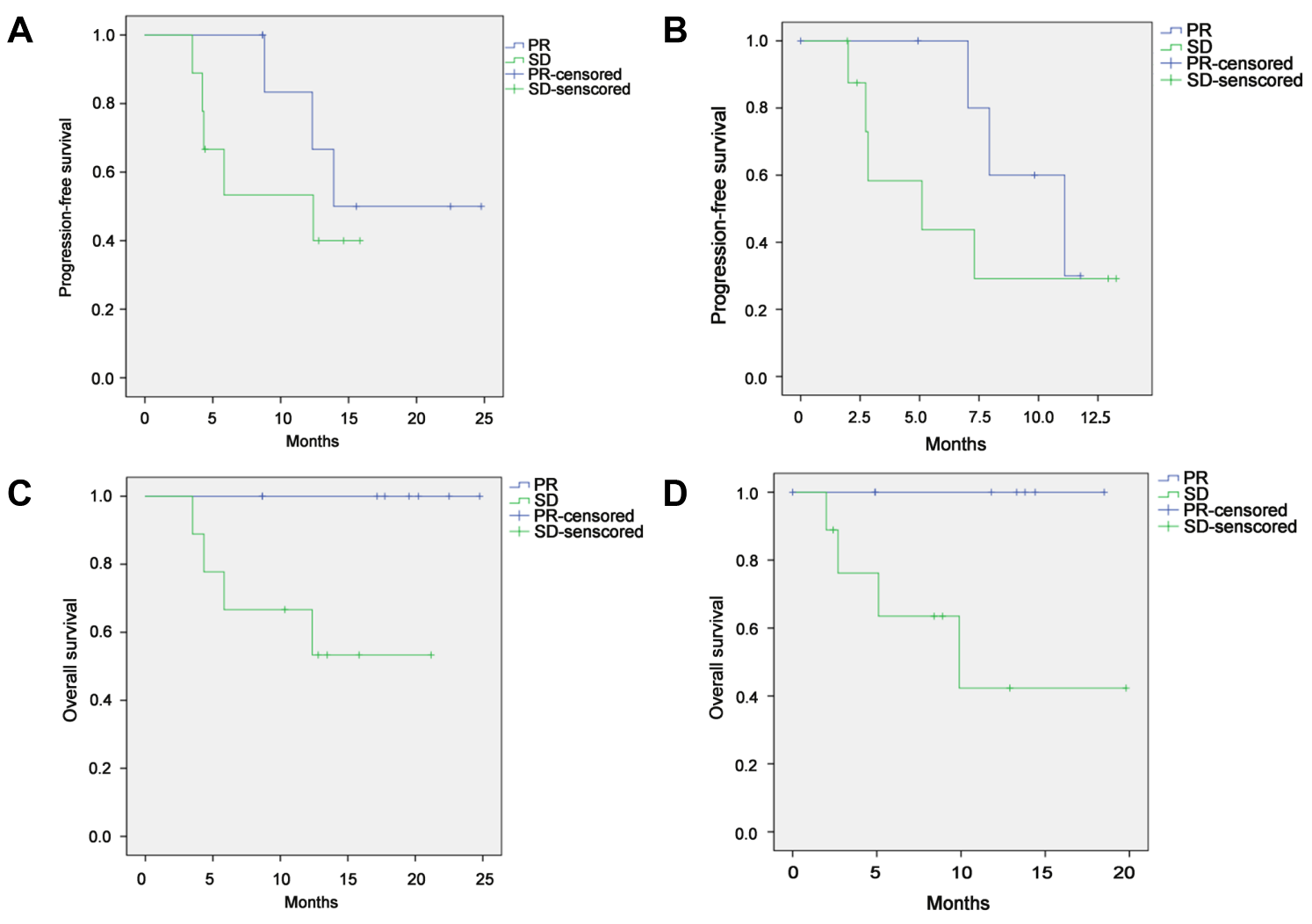


**Supplementary Figure S4.** Comparisons of progression-free survival (PFS) and overall survival (OS) in patients with different responses before discontinuation. (A) PFS from the treatment. (B) PFS from the discontinuation. (C) OS from the treatment. (D) OS from the discontinuation.


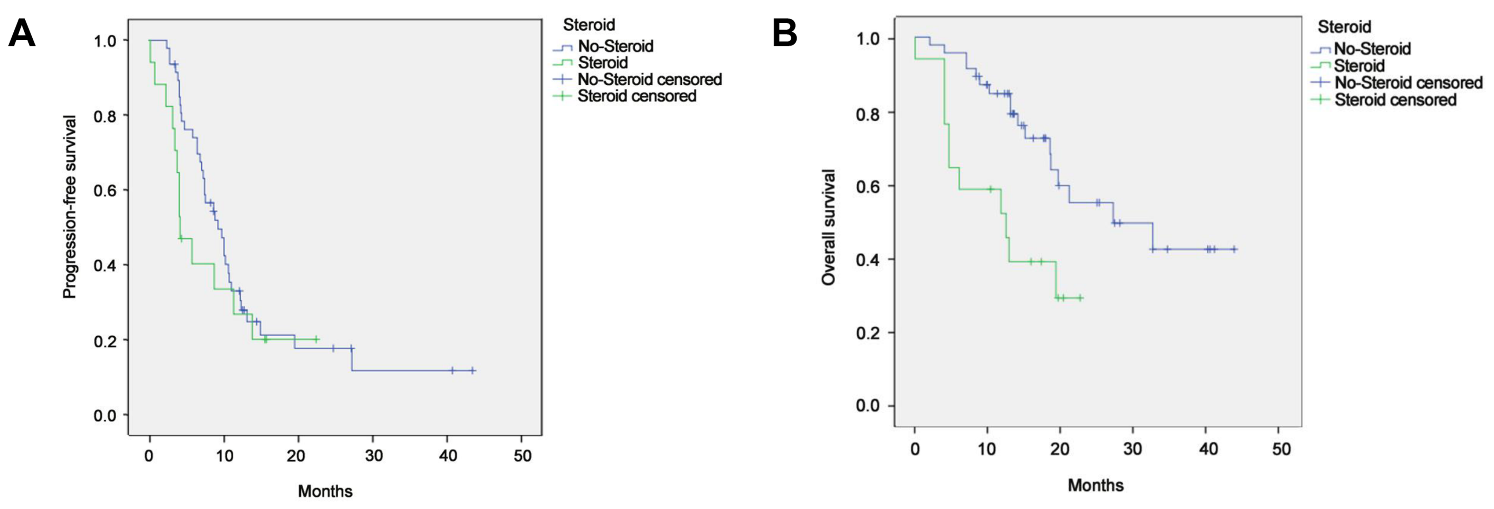


**Supplementary Figure S5.** Comparisons of progression-free survival (PFS) and overall survival (OS) in irAE patients who were treated or not with steroids.


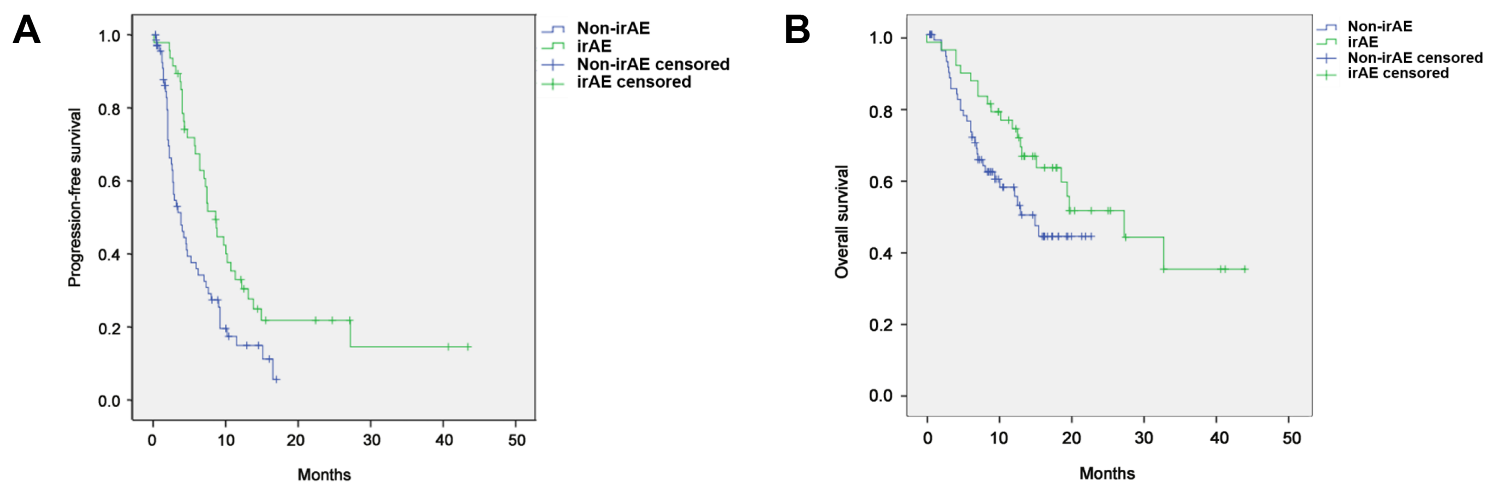


**Supplementary** **Figure S6.** Subgroup analysis of stage IV patients. Among the 118 stage IV patients, 47 were in the irAE group, and 71 were in the no-irAE group. (A) The median progression-free survival (PFS) was 8.7 (95% CI: 7.0-10.4) months in the irAE group and 3.9 (95% CI: 2.4-5.4) months in the non-irAE group (P=0.002). (B) The median overall survival (OS) was 27.0 (95%CI: 13.4-40.6) months in the irAE group and 14.8 (95%CI: 10.7-18.9) months in the non-irAE group (P=0.069).

## Supplementary Tables

**Supplementary Table S1.** Treatment of PD-1 and PD-L1 inhibitors

| Drug | Single drug (n=99, 51.8%) | Combined with chemotherapy (n=82, 42.9%) | Combined with CTLA-4 (n=6, 3.1%) | Others (n=4, 2%) |
| --- | --- | --- | --- | --- |
| Pembrolizumab | 11 (1-10) | 39 (1-6) | 0 | 4 (2-6) |
| Nivolumab | 43 (1-19) | 19 (1-12) | 0 | 0 |
| Durvalumab | 5 (1-24) | 0 | 6 (1-18) | 0 |
| Atezolizumab | 17 (1-47) | 6 (3-19) | 0 | 0 |
| Avelumab | 3 (2-35) | 0 | 0 | 0 |
| IBI308 | 4 (1-3) | 12 (1-12) | 0 | 0 |
| SHR1210 | 1 (2) | 6 (1-26) | 0 | 0 |
| BGB-A317 | 8 (1-9) | 0 | 0 | 0 |
| CS1001 | 1 (15) | 0 | 0 | 0 |
| Others | 6 (1-5) | 0 | 0 | 0 |

PD-1: programmed cell death protein 1; PD-L1: programmed cell death protein ligand 1; CTLA-4: cytotoxic T-lymphocyte-associated protein 4.
